# The Apoptotic Resistance of BRCA1-Deficient Ovarian Cancer Cells is Mediated by cAMP

**DOI:** 10.3389/fcell.2022.889656

**Published:** 2022-04-20

**Authors:** Wei Yue, Jihong Ma, Yinan Xiao, Pan Wang, Xiaoyang Gu, Bingteng Xie, Mo Li

**Affiliations:** ^1^ Center for Reproductive Medicine, Department of Obstetrics and Gynecology, Peking University Third Hospital, Beijing, China; ^2^ National Clinical Research Center for Obstetrics and Gynecology (Peking University Third Hospital), Beijing, China; ^3^ Key Laboratory of Assisted Reproduction (Peking University), Ministry of Education, Beijing, China; ^4^ Beijing Key Laboratory of Reproductive Endocrinology and Assisted Reproductive Technology (Peking University Third Hospital), Beijing, China; ^5^ School of Life Science, Beijing Institute of Technology, Beijing, China

**Keywords:** BRCA1-deficient tumor, ADRB1, cAMP, apoptosis, immune suppression

## Abstract

Breast cancer type 1 susceptibility protein (BRCA1) is essential for homologous recombination repair of DNA double-strand breaks. Loss of BRCA1 is lethal to embryos due to extreme genomic instability and the activation of p53-dependent apoptosis. However, the apoptosis is resisted in BRCA1-deficient cancer cells even though their p53 is proficient. In this study, by analysis of transcriptome data of ovarian cancer patients bearing BRCA1 defects in TCGA database, we found that cAMP signaling pathway was significantly activated. Experimentally, we found that BRCA1 deficiency caused an increased expression of ADRB1, a transmembrane receptor that can promote the generation of cAMP. The elevated cAMP not only inhibited DNA damage-induced apoptosis through abrogating p53 accumulation, but also suppressed the proliferation of cytotoxic T lymphocytes by enhancing the expression of immunosuppressive factors DKK1. Inhibition of ADRB1 effectively killed cancer cells by abolishing the apoptotic resistance. These findings uncover a novel mechanism of apoptotic resistance in BRCA1-deficient ovarian cancer cells and point to a potentially new strategy for treating BRCA1-mutated tumors.

## Introduction

As a core homologous recombination (HR) factor, BRCA1 functions in the maintenance of genome integrity (Deng and Brodie, 2000; Huen et al., 2010; Tarsounas and Sung, 2020). Loss of BRCA1 causes a failure of repairing DNA double-strand breaks (DSBs) and leads to the accumulation of DNA lesions in cells (Eyfjord and Bodvarsdottir, 2005; Savage and Harkin, 2015). It has been reported that BRCA1 deficiency in mice causes early embryonic death before day E7.5 because of extreme genomic instability and p53-dependent apoptosis activation (Gowen et al., 1996; Hakem et al., 1996; Ludwig et al., 1997). BRCA1 null primary mouse embryonic fibroblast cells display severe growth arrest phenotypes and also activation of p53-depende nt apoptosis (Xu et al., 2001; Cao et al., 2006; Drost et al., 2011; Zhu et al., 2011). On the other hand, women carrying *BRCA1* mutations have a 50%–80% risk of developing breast cancer and a 40%–65% risk of developing ovarian cancer during their lifetime (Eastson et al., 1995; Rahman and Stratton, 1998; King et al., 2003). Interestingly, these *BRCA1*-mutated cancer cells resist apoptosis and proliferate smoothly (Elledge and Amon, 2002; Monteiro, 2003). This paradox can be partially explained by the coexistence of *p53* mutations which abrogates p53-dependent apoptosis (Brodie and Deng, 2001; Aubrey et al., 2018; Hafner et al., 2019). However, a considerable proportion of cancer cells from BRCA1-deficient breast or ovarian cancer patients bear no p53 mutations (Ramus et al., 1999; Greenblatt et al., 2001; Manie et al., 2009; Jonsson et al., 2019). Thus, these cancer cells may evolve unknown abilities for tumor survival.

Apoptosis is a programmed cell death process that can be triggered by multiple stresses, such as DNA damage, cytotoxic chemicals, and oxidative stress. Apoptosis is essential for the elimination of genome-unstable cells and the maintenance of homeostasis (Carneiro and El-Deiry, 2020). However, various tumor types have developed specific approaches to alter apoptotic pathways, leading to defects in apoptosis (Ghobrial et al., 2005). Exploring the specific pathways that resist apoptosis in a certain tumor will be conducive to discover novel targeted drugs. Recent investigations reveal that cAMP participates in promoting cancer cell proliferation, migration, invasion, and metabolism, and is a potential apoptotic suppressor (Naderi et al., 2009; Sood et al., 2010; Creed et al., 2015; Pon et al., 2016; Zhang et al., 2019). As an intracellular second messenger, cAMP performs signal transduction roles in many biological processes, such as gene expression regulation, neurotransmitter synthesis, and cell metabolism (Yan et al., 2016; Patra et al., 2021). Of note, many proteins that can promote the generation of cAMP are up-regulated in cancers (Sales et al., 2001; Chang et al., 2004; Lehrer and Rheinstein, 2020).

In this study, by analyzing the transcriptome data from TCGA database, we found that cAMP signaling pathway was significantly activated in BRCA1-defective ovarian cancer patients. In addition, genes that involved in regulating this pathway, such as ADRB1, a β-adrenocepter that can promote the production of cAMP, were up-regulated in BRCA1-defective ovarian cancer patients. When BRCA1 was knocked down in ovarian cancer cell lines bearing wide-type BRCA1, the expression of ADRB1 was significantly increased. ADRB1 enhanced the level of cAMP in BRCA1 knock-down cells that resisted p53-dependent apoptosis induced by DNA damage. Moreover, cAMP could also induce the expression of DDK1, which is a secreted factor that can suppress the cytotoxic T lymphocytes to kill cancer cells. Inhibition of ADRB1 by its selective inhibitor abrogated its ability to inhibit p53-dependent apoptosis. In conclusion, our study uncovers an underlying mechanism by which BRCA1-deficient cancer cells resist apoptosis, and identifies possible therapeutic targets for *BRCA1*-mutated tumors.

## Materials and Methods

### Bioinformatics Analysis

The integrated dataset containing clinical information, BRCA1 mutation information, transcriptome data of 594 ovarian cancer patients (TCGA, Firehose Legacy) was acquired from the cBioPortal database (https://www.cbioportal.org/). The overall survival analysis between BRCA1-deficient and -proficient group, differentially expressed genes analysis between BRCA1-deficient and -proficient group, gene expression correlation analysis between BRCA1 and DNA damage repair genes, and gene mutation frequency analysis between BRCA1-deficient and -proficient group were conducted using online tool in cBioPortal (Cerami et al., 2012; Gao et al., 2013). Gene Ontology (GO) and Kyoto Encyclopedia of Genes and Genomes (KEGG) enrichment analysis of up-regulated and down-regulated genes were performed by using the WebGestalt (http://www.webgestalt.org/#) (Liao et al., 2019). Gene set enrichment analysis (GSEA) was performed based on the normalized mRNA expression data (RNA Seq V2 RSEM) using GSEA software with default setting (http://www.broadinstitute.org/gsea) (Mootha et al., 2003; Subramanian et al., 2005). Normalized enrichment score (NES) and false discovery rate (FDR) of each gene sets were calculated.

### Chemicals and Antibodies

All chemicals were purchased from Sigma except for those specifically mentioned. The CFSE (carboxyfluorescein succinimidyl ester, HY-D0938), dobutamine (HY-15746), atenolol (HY-17498), and epinephrine (HY-B0447B) were purchased from MCE. The 8-CPT-cAMP (BML-CN130-0020) was purchased from LDBIO. Anti-β-actin (66009-1-lg) antibody was purchased from Proteintech. Anti-cleaved Caspase-3 (Asp175) 9664 antibody was purchased from Cell Signaling Technology. Anti-Bax (6A7) (sc-23959) antibody was purchased from Santa Cruz Biotechnology. Anti-ADRB1 (ab85037) and Anti-DKK1 (ab93017) antibody was purchased from Abcam. Anti-P53 (NB200-103) antibody was purchased from Novus. Anti-BRCA1 (PA5-88149) antibody, Alexa Fluor™ 647 Phalloidin (A22287), Alexa FluorTM 488 goat anti-mouse IgG (A-11001), HRP goat anti-mouse IgG (H + L) secondary antibody (32430), and HRP goat anti-rabbit IgG (H + L) secondary antibody 31466) were purchased from Thermo Fisher Scientific.

### Plasmid Construction

To knockdown the expression of *BRCA1*, oligos encoding *BRCA1* shRNA was cloned into pLKO.1 plasmid. shRNA sequence was designed using “shRNAs for Individual Genes” purchased from Sigma Aldrich. The sequences of negative control (NC) and gene targeting shRNA were provided in Supplementary Table S2.

### Cell Culture, Chemicals Treatment, and IR Treatment

HEK-293T and A2780 cells were cultured in DMEM medium supplemented with 10% fetal bovine serum (FBS), and 100 U/ml penicillin-streptomycin in a 37°C incubator with 5% CO_2_. OVCAR-5 and IGROV-1 cells were cultured in RPMI-1640 medium supplemented with 10% fetal bovine serum (FBS), and 100 U/ml penicillin-streptomycin in a 37°C incubator with 5% CO_2_. For chemicals treatment, cells were incubated with epinephrine (10 μM), dobutamine (10 μM), atenolol (50 μM), ICI-118551 (50 μM), or 8-CPT-cAMP (200 μM) for 90 min before ELISA experiments or IR treatment. For IR treatment, cells were irradiated with a 137Cs source at a dose of 10 Gy. After 18 h, the cells were used for RTCA, flow cytometry, immunofluorescence, and western blot experiments.

### Immunofluorescence

Cells were fixed in 4% paraformaldehyde in PBS (pH 7.4) for 30 min followed by permeabilization with PBS containing 0.5% Triton-X-100 for 25 min at room temperature. Cells were blocked with 1% bovine serum albumin-supplemented PBS for 1 h and then incubated with the indicated primary antibodies (1:200–1:500) diluted in 3% bovine serum albumin-supplemented PBS at 4°C overnight. After washing three times in PBS containing 0.1% Tween 20 and 0.01% Triton-X 100, cells were incubated with an appropriate fluorescent secondary antibody for 1 h at room temperature. After washing three times, samples’ nuclear were stained with Hoechst 33342 (10 μg/ml) for 10 min and subsequently mounted on glass slides. Images were acquired using a confocal laser scanning microscope with a 63 x/1.40 oil objective (Carl Zeiss 880).

### shRNA Lentivirus Generation and shRNA Knockdown

For shRNA lentivirus generation, the pLKO.1 plasmid comprising shRNA was co-transfected with the packaging plasmids (psPAX2 and pMD2. G) into HEK293T cells using Lipofectamine 3000™ according to the manufacturer’s protocol. Six hours after transfection, the cells were washed and changed with fresh growth culture media and incubated for another 48 h. Then the culture media containing viral particles were harvested and centrifuged at 3,000 ×*g* for 5 min to remove the cell debris and filtered by a 0.45-μm filter. The viral supernatant was further concentrated with a Centricon Plus-20 Centrifugal Filter at 4,000 ×*g*. The concentrated lentivirus supernatant was aliquoted and kept at -80°C before use. To knock down *BRCA1* mRNA in indicated cells, 1 × 10^5^ cells were seeded onto 6-well plates and incubated at 37°C with 5% CO_2_ until reaching 30–40% confluence. The concentrated viral supernatant was added into the culture medium at a multiplicity of infection (MOI) of 20. After 72 h, puromycin was added to the medium at 1 μg/ml for stable knock-down selection.

### Western Blot

Total protein was extracted from cell lysate by RIPA buffer. Protein samples were separated by sodium dodecyl sulfate polyacrylamide gel electrophoresis (SDS-PAGE) and then electrically transferred to polyvinylidene fluoride membranes. Following transfer, the membranes were blocked in TBST containing 5% skim milk for 1 h at room temperature, and then incubated with primary antibodies (1:500–1:1,000 dilution) overnight at 4°C. After washing in TBST three times, the membranes were incubated at 37°C for 1 h with a 1:1,000 dilution of HRP-conjugated secondary antibody. Finally, protein bands were visualized using an enhanced chemiluminescence detection system (Amersham Biosciences).

### cAMP ELISA

cAMP level was measured with Human cAMP ELISA Kit (Sino BestBio, CK-E10885). In total, 5 × 10^6^ cells were harvested, washed with PBS and lysed in 500 μL RIPA Lysis buffer (Pierce, 89900) on ice for 20 min. Then the samples were centrifuged at 1000 ×*g* at 4°C for 15 min, and the supernatant was used to measure cAMP concentration according to the manufacturer’s protocol.

### RT-qPCR

The total RNA of tumor cells was extracted by TRIzol reagent (Gibco, 15596026) and 2 μg RNA of each sample was reverse transcribed into cDNA with RevertAid RT Reverse Transcription Kit (Thermo, K1691). RT-qPCR was performed on the StepOnePlus system (ABI) with PowerUp™ SYBR™ Green Master Mix (Thermo, A25742). Conditions of RT-qPCR were 95°C for 2 min; 95°C for 3 s and 60°C for 30 s for 40 cycles. Relative expression values of each target genes were normalized to mRNA expression of the housekeeping gene GAPDH. The relative mRNA expression level was calculated through the comparative cycle threshold method (2^−ΔΔCt^). The primers were provided in Supplementary Table S2.

### Cell Proliferation Assay by xCELLigence RTCA System

Cell proliferation was assessed using the xCELLigence RTCA system (Acea Bioscience, San Diego, CA, United States, distributed by Roche Diagnostics) that allows long-term monitoring of live cells in a noninvasive manner (Heinecke et al., 2014; Al Nakouzi et al., 2016). In brief, 5,000–10,000 cells were seeded in each well of E-16-well plates (Roche). Cell proliferation was monitored for 40–70 h at 37°C in the incubator. Microelectrodes on the bottom of plates were used to detect impedance changes proportional to the number of adherent cells. The impedance value of each well was automatically recorded by Real-Time Cell Analyzer (RTCA) software. Two parallel wells were included for each sample in one replicate, and three independent replicates were conducted.

### CD8^+^ T Cells Proliferation Assay

Peripheral blood mononuclear cells (PBMCs) of human were obtained from healthy volunteers and used for isolation of CD8^+^ T cells. The CD8^+^ T cells were selected using the MagniSort™ Human CD8^+^ T cell Enrichment Kit (Thermo fisher, 8804-6812-74) according to the manufacturer’s protocol. 2 × 10^5^ isolated cells were labeled with CFSE and cultured in the 96-well plate. After incubated with Human T-Activator CD3/CD28 Dynabeads (Thermo fisher, 11161D) for 3 days, the CD8^+^ T cells were activated to proliferate. The activated CD8^+^ T cells were continuously co-cultured with the culture supernatant for 3 days. Then, the cells were collected and analyzed by flow cytometry.

### Flow Cytometry

For cell cycle analysis, cells were washed with PBS, trypsinized, and centrifuged at 1,500 rpm for 3 min. Then the cells were washed three times with 1% BSA in PBS at 1,500 rpm for 3 min followed by fixation in 70% ethanol at 4°C overnight. The fixed cells were washed with ice-cold PBS twice and incubated with RNaseA (50 μg/ml) at 37°C for 30 min. After staining with PI (10 μg/ml) for 30 min, a total of 10,000 cells of each sample was analyzed by a FACSCalibur™ Flow Cytometer (BD, Franklin Lakes, NJ, United States) and the data were analyzed using FlowJo software. Cell apoptosis analysis was performed using Annexin V-FITC/PI Apoptosis Detection Kit (Vazyme, Nanjing, China). Briefly, cells were washed with PBS, trypsinized, and centrifuged at 1,500 rpm for 3 min. Then the cells were washed with PBS followed by staining with annexin V-FITC and propidium iodide, a total of 10,000 cells of each sample was analyzed using a BD FACScan flow cytometry system (Becton Dickinson, Franklin, NJ, United States).

### Statistical Analyses

All experiments were performed in triplicate unless indicated otherwise. Means and standard deviations were plotted. Student’s *t*-test was used for statistical analyses. *p* < 0.05 was considered statistically significant. Statistical details are showed in figure legends.

## Results

### BRCA1 Deficiency is Associated With Poor Survival Outcomes in Ovarian Cancer Patients

**FIGURE 1:**
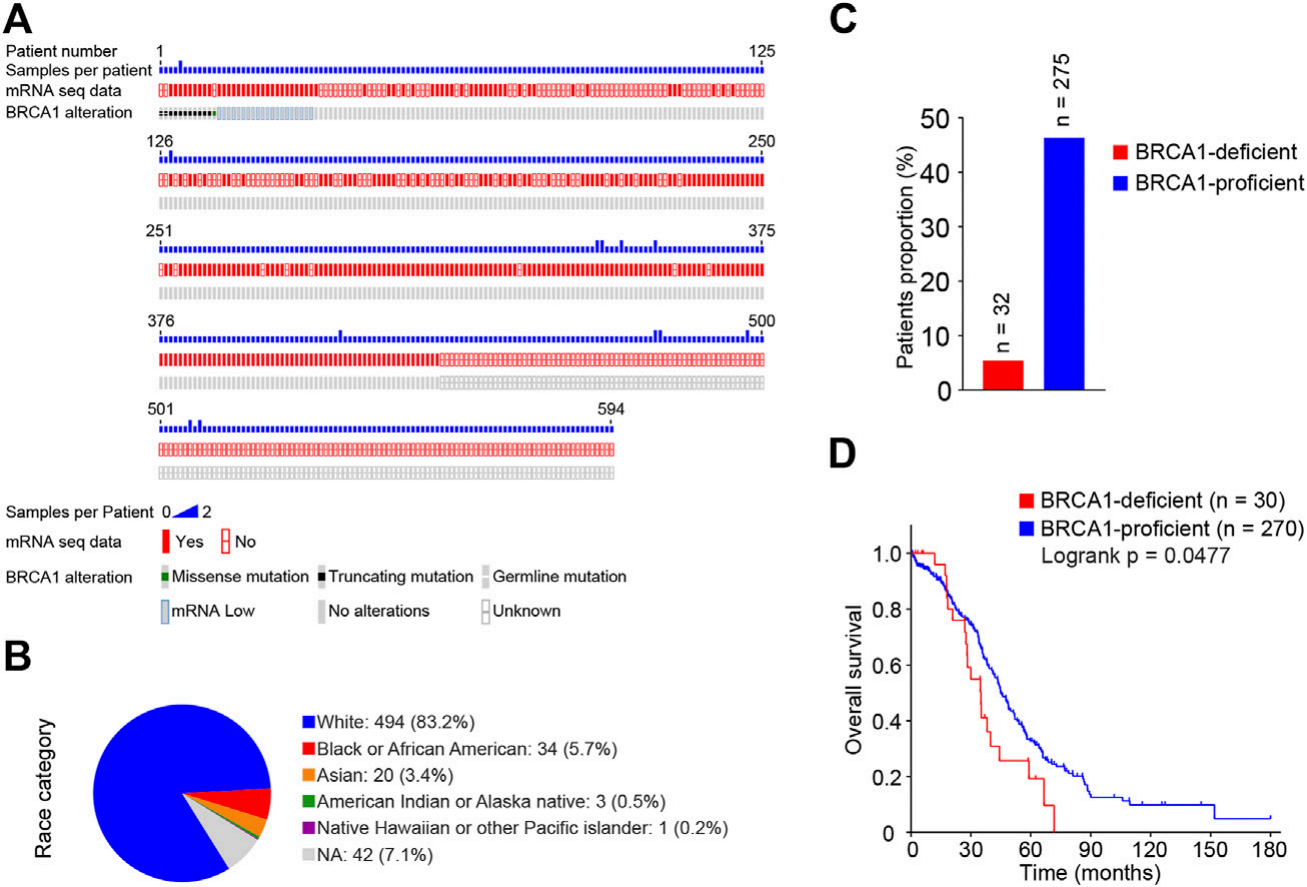
Survival analysis of BRCA1-deficient ovarian cancer patients from TCGA database. **(A)** *BRCA1* mutations and mRNA transcriptome information plot of ovarian cancer patients from TCGA database downloaded from the cBioPortal. A total of 606 samples from 594 ovarian cancer patients in the dataset were recorded. **(B)** Race category of ovarian cancer patients from **(A)**. Of the 594 patients 83.2% were Caucasian, 5.7% were African American, 3.4% were Asian. **(C)** Proportions of BRCA1-deficient (mutated and mRNA low) and -proficient (wild-type and mRNA normal) patients. 32 BRCA1-deficient, 275 BRCA1-proficient were included. **(D)** Kaplan-Meier survival curve of overall survival between BRCA1-deficient patients (*n* = 30, red) and BRCA1-proficient patients (*n* = 270, blue). Patients with related clinical information were included. LogRank *p* = 0.0477.

Through the cBioPortal database (https://www.cbioportal.org/), we obtained clinical information integrated with genome and transcriptome data for ovarian cancer patients [Ovarian Serous Cystadenocarcinoma (TCGA, Firehose Legacy)] (Cerami et al., 2012; Gao et al., 2013). A total of 606 tumor tissue samples from 594 patients, all with serous ovarian cancers, were recorded in this dataset (Figure 1A, Supplementary Table S1). Of the 594 patients 83.2% were Caucasian, 5.7% were African American, and 3.4% were Asian (Figure 1B). We found 12 patients with *BRCA1* mutations based on the genome sequence data, and each of them carried one type of BRCA1 mutation. Another 20 patients carrying a wild-type *BRCA1* gene had low levels of BRCA1 mRNA. Therefore, we summarized the 32 cases with defective BRCA1 function (BRCA1-deficient group); 29 of them had corresponding transcriptome data. A total of 275 cases carried the wild-type *BRCA1* gene and expressed normal levels of BRCA1 (BRCA1-proficient group) (Figures 1A,C). Next, we compared the survival status of ovarian cancer patients with or without BRCA1 deficiency. As shown in Figure 1D, cancer patients with defective BRCA1 had significantly shorter overall survival outcomes than those with normal BRCA1. This indicates that BRCA1 deficiency predicts poor outcomes for ovarian cancer patients.

### BRCA1 Deficiency Impairs DNA Damage Repair in Ovarian Cancer Patients

**FIGURE 2:**
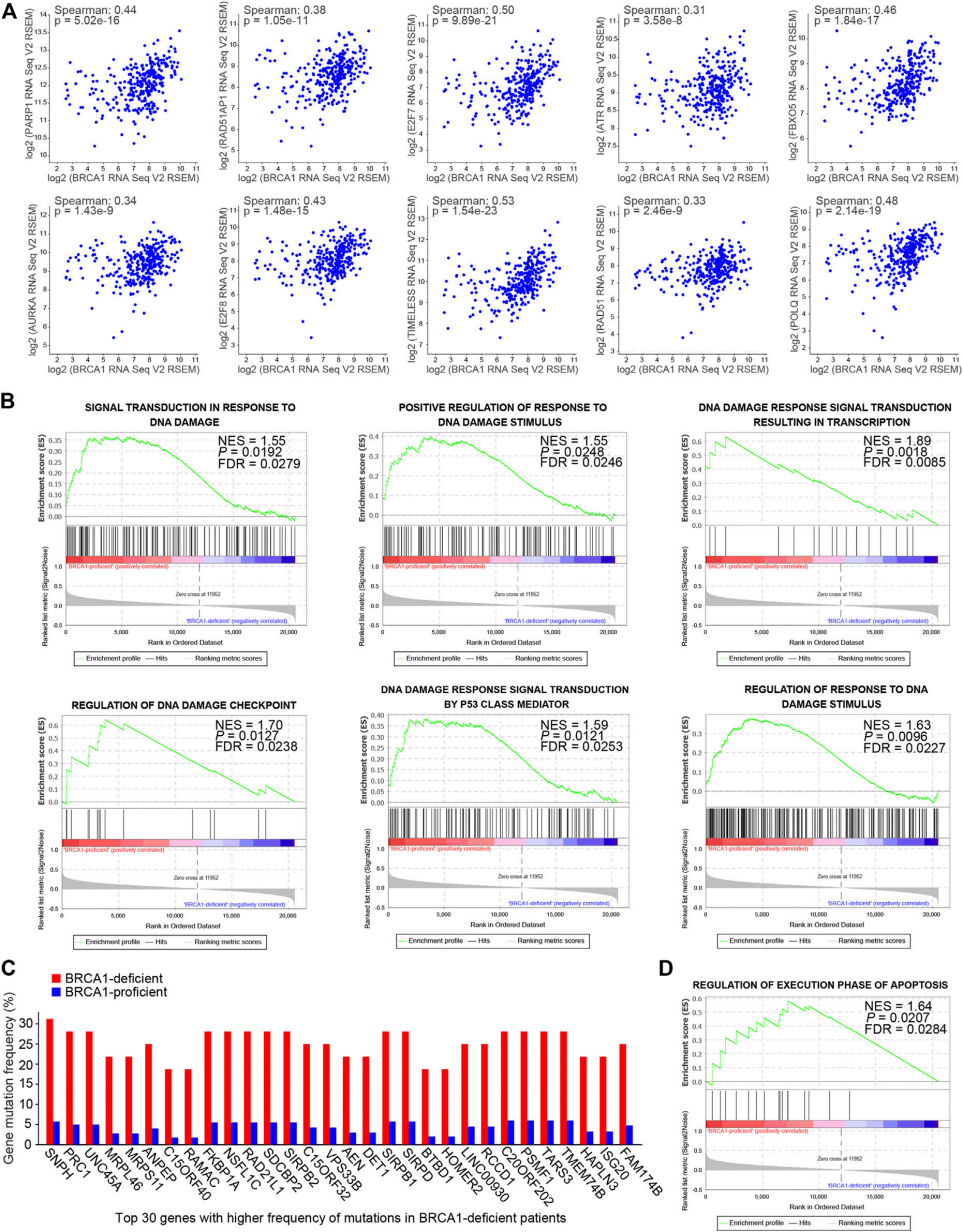
DNA damage responses were compromised in BRCA1-deficient ovarian cancer patients. **(A)** mRNA expression correlation analysis of the *BRCA1* gene and genes related to DNA damage response among the ovarian cancer patients. *BRCA1* gene expression was positively correlated with these genes. Spearman: spearman correlation coefficient. **(B)** GSEA analysis of gene sets related to DNA damage responses based on the normalized mRNA expression data (RNA Seq V2 RSEM) between BRCA1-proficient (*n* = 275) and -deficient (*n* = 29) patients. NES, normalized enrichment score. FDR, false discovery rate. Positive NES indicates lower expression in BRCA1-deficient patients. **(C)** Gene mutation frequency analysis between BRCA1-deficient patients (red) and -proficient patients (blue). Top 30 genes with significantly higher frequency of mutations in BRCA1-deficient patients compared with BRCA1-proficient patients are shown. **(D)** GSEA analyses of gene set related to regulation of execution phase of apoptosis based on the normalized mRNA expression data (RNA Seq V2 RSEM) between BRCA1-proficient (*n* = 275) and -deficient (*n* = 29) patients. NES, normalized enrichment score. FDR, false discovery rate. Positive NES indicates lower expression in BRCA1-deficient patients.

Using the transcriptome data, we analyzed the correlation in mRNA expression levels of the *BRCA1* gene and genes related to DNA damage response among the ovarian cancer patients. We found that BRCA1 expression was positively correlated with that of each of the DNA damage responsive genes tested: *PARP1*, *RAD51AP1*, *E2F7*, *ATR*, *FBXO5*, *AURKA*, *E2F8*, *TIMELESS*, *RAD51*, and *POLQ* (Figure 2A). The results suggest that BRCA1 deficiency may cause inadequate DNA damage repair due to the lack of DNA damage repair factors in ovarian cancer cells.

To explore the effects of BRCA1 deficiency in the ovarian cancer patients, we analyzed mRNA expression differences between the BRCA1-deficient and -proficient groups using Gene set enrichment analysis (GSEA) (Mootha et al., 2003; Subramanian et al., 2005). As shown in Figure 2B, gene sets related to DNA damage responses were enriched in the BRCA1-proficient group, including signal transduction in response to DNA damage (NES = 1.55), positive regulation of response to DNA damage stimulus (NES = 1.55), DNA damage response signal transduction resulting in transcription (NES = 1.89), regulation of DNA damage checkpoint (NES = 1.70), DNA damage response signal transduction by p53 class mediator (NES = 1.59), and regulation of response to DNA damage stimulus (NES = 1.63). The positive NES value indicates that genes in these gene sets are expressed lower in BRCA1-deficient patients than in BRCA1-proficient patients, which suggests that responses to DNA damage are attenuated in ovarian cancer cells with defective BRCA1. The lack or deficiency of DNA damage repair inevitably causes gene mutations, which are sources of genome instability in cancer cells. Therefore, we further analyzed the gene mutations within the BRCA1-defective ovarian cancer patients. As expected, the gene mutation frequency in the BRCA1-deficient patients was much higher than that in patients with normal BRCA1 (Figure 2C). However, extreme genome instability caused by these mutations did not result in more obvious apoptosis in the BRCA1-defective ovarian cancer patients than that in patients with normal BRCA1 (Figure 2D). These results indicate that BRCA1-deficient cancer cells may have mechanisms that allow them to resist apoptosis, even in the presence of persistent DNA damage and extreme genome instability.

### cAMP Signaling is Significantly Activated in the BRCA1-Deficient Ovarian Cancer Patients

**FIGURE 3:**
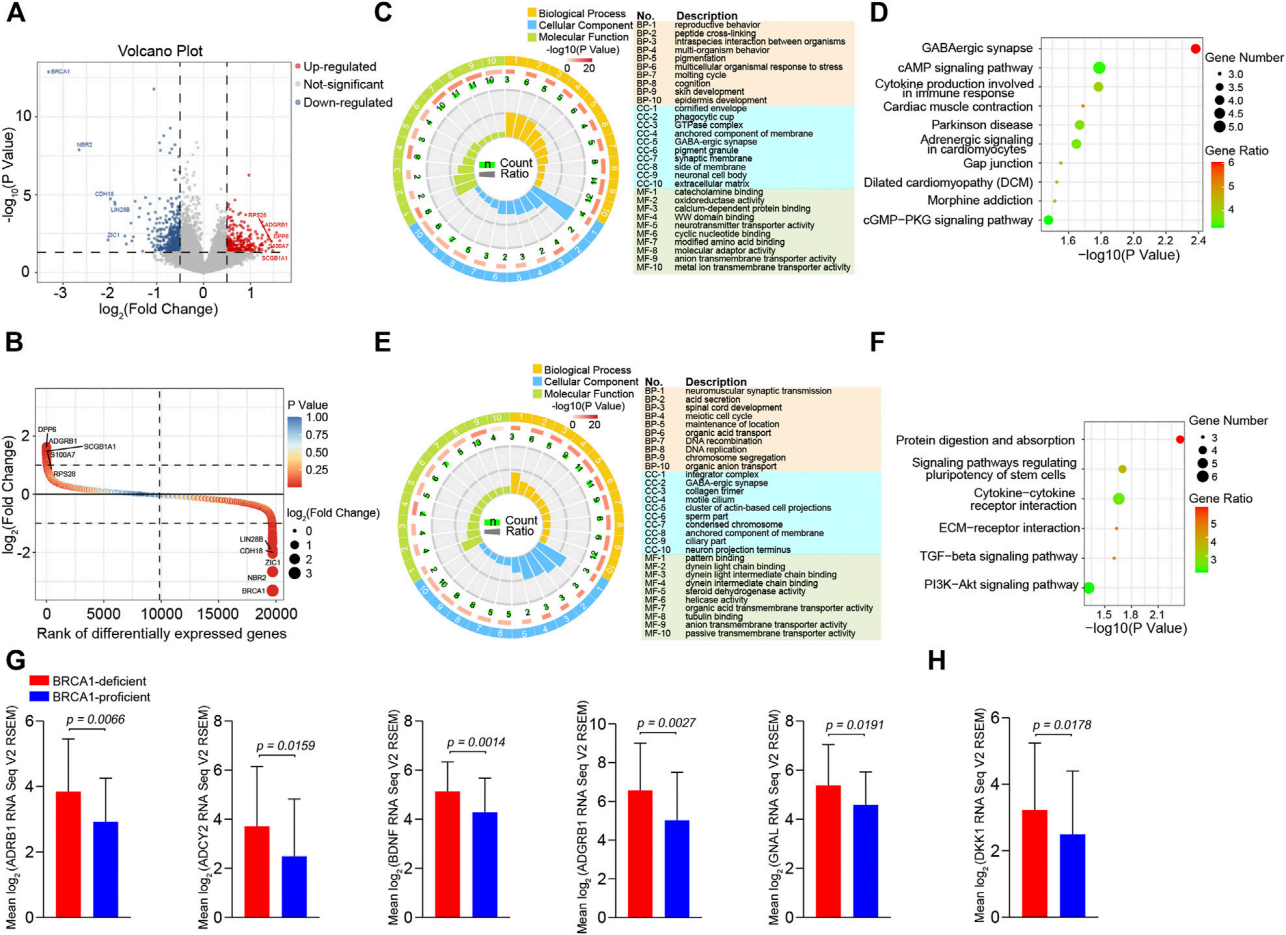
Differentially expressed genes analysis between BRCA1-deficient and BRCA1-proficient ovarian cancer patients. **(A)** Volcano plot of differentially expressed genes (fold change >1.5, *p* value <0.05) between BRCA1-deficient (*n* = 29) and -proficient (*n* = 275) patients. In total, 447 differentially expressed genes including 176 up-regulated genes (red) and 271 down-regulated genes (blue) were identified in BRCA1-deficient ovarian cancer patients. **(B)** Rank plot of differentially expressed genes according to gene expressing difference. The highest five up-regulated and down-regulated genes in BRCA1-deficient patients are labelled in the left and right in the plot, respectively. **(C)** GO enrichment analysis of 176 up-regulated genes. The first lap indicates top 10 GO terms in “biological process,” “cellular component,” and “molecular function,” the description of each item is listed in the right. *p* value and the number of the genes for corresponding GO terms are shown in the second and the third laps, respectively. Enrichment factors of each GO term are shown in the fourth lap. **(D)** KEGG analysis of 176 up-regulated genes. Top 10 most significantly pathways are shown in the plot. The color key from green to red represents the gene ratio. Dot size indicates the number of the genes in corresponding pathway. **(E)** GO enrichment analysis of 271 down-regulated genes. The first lap indicates top 10 GO terms in “biological process,” “cellular component,” and “molecular function,” the description of each item is listed in the right. *p* value and the number of the genes for corresponding GO terms are shown in the second and the third laps, respectively. Enrichment factors of each GO term are shown in the fourth lap. **(F)** KEGG analysis of 271 down-regulated genes. Top six most significantly pathways are shown in the plot. The color key from green to red represents the gene ratio. Dot size indicates the number of the genes in corresponding pathway. **(G)** Comparison of *ADRB1*, *ADCY2*, *BDNF*, *ADGRB1*, and *GNAL* expression between BRCA1-deficient (*n* = 29, red) and -proficient (*n* = 275, blue) patients. **(H)** Comparison of *DKK1* expression between BRCA1-deficient (*n* = 29, red) and -proficient (*n* = 275, blue) patients.

To uncover the mechanism underlying the apoptotic resistance of BRCA1-deficient ovarian cancer cells, we analyzed the differentially expressed genes (DEGs) between the BRCA1-deficient and -proficient cases. We identified 447 DEGs (fold change >1.5, *p* value <0.05), 176 up-regulated and 271 down-regulated, in the BRCA1-deficient ovarian cancer patients (Figure 3A). Also, we ranked the DEGs according to difference in gene expression level (Figure 3B). The most significantly up-regulated genes were *DPP6*, *ADGRB1*, *SCGB1A1*, *S100A7*, and *RPS28*, and the most significantly down-regulated genes were *BRCA1*, *NBR2*, *ZIC1*, *CDH18*, and *LIN28B*. Based on these results, we can see that BRCA1-deficient ovarian cancer cells expressed lower levels of BRCA1 mRNA as expected.

Next, we performed Gene Ontology (GO) and Kyoto Encyclopedia of Genes and Genomes (KEGG) pathway enrichment analysis of the DEGs. The main enriched molecular function terms for the up-regulated genes in BRCA1-defective ovarian cancer cells were “catecholamine binding,” “oxidoreductase activity, acting on the CH-NH group of donors,” “calcium-dependent protein binding,” “WW domain binding,” “neurotransmitter transporter activity,” and “cyclic nucleotide binding” (Figure 3C). KEGG analysis showed that the most significantly enriched pathways were “GABAergic synapse,” “cAMP signaling pathway,” “Cytokine production involved in immune response,” and “Adrenergic signaling in cardiomyocytes” (Figure 3D). In the down-regulated gene analysis, molecular functions associated with “pattern binding,” “dynein light chain binding,” “steroid dehydrogenase activity,” “helicase activity,” and “organic acid transmembrane transporter activity,” were enriched (Figure 3E). The “Protein digestion and absorption,” “Signaling pathways regulating pluripotency of stem cells,” “Cytokine-cytokine receptor interaction,” and “ECM-receptor interaction” KEGG pathways were obviously enriched (Figure 3F). When inspecting the results above, we particularly noticed that genes involved in the ADRB1-mediated cAMP signaling pathway were dramatically up-regulated in BRCA1-deficient ovarian cancer patients (Figures 3D,G). In addition, genes that participate in regulating this pathway, including those involved in “catecholamine binding,” “calcium-dependent protein binding,” and “cyclic nucleotide binding,” were also dramatically up-regulated (Figure 3C). These data demonstrate that cAMP signaling is significantly activated in BRCA1-deficient ovarian cancer patients. A series of studies have illustrated that cAMP can inhibit cancer cell apoptosis induced by DNA damage (Nishihara et al., 2003; Naderi et al., 2009). Besides, the expression of DKK1 in the “cytokine production involved in immune response” pathway was also elevated in BRCA1-deficient ovarian cancer patients (Figures 3D,H). DKK1 is a secreted factor that has immune inhibitory effects through suppressing the proliferation of CD8^+^ T cells and NK cells, thus leading to immune evasion of cancer cells (Chu et al., 2021). Therefore, BRCA1-deficient ovarian cancer cells may develop two ways to resist cell death.

### BRCA1 Knock-Down Induces Elevated Expression of ADRB1 for an Increased Generation of cAMP

**FIGURE 4:**
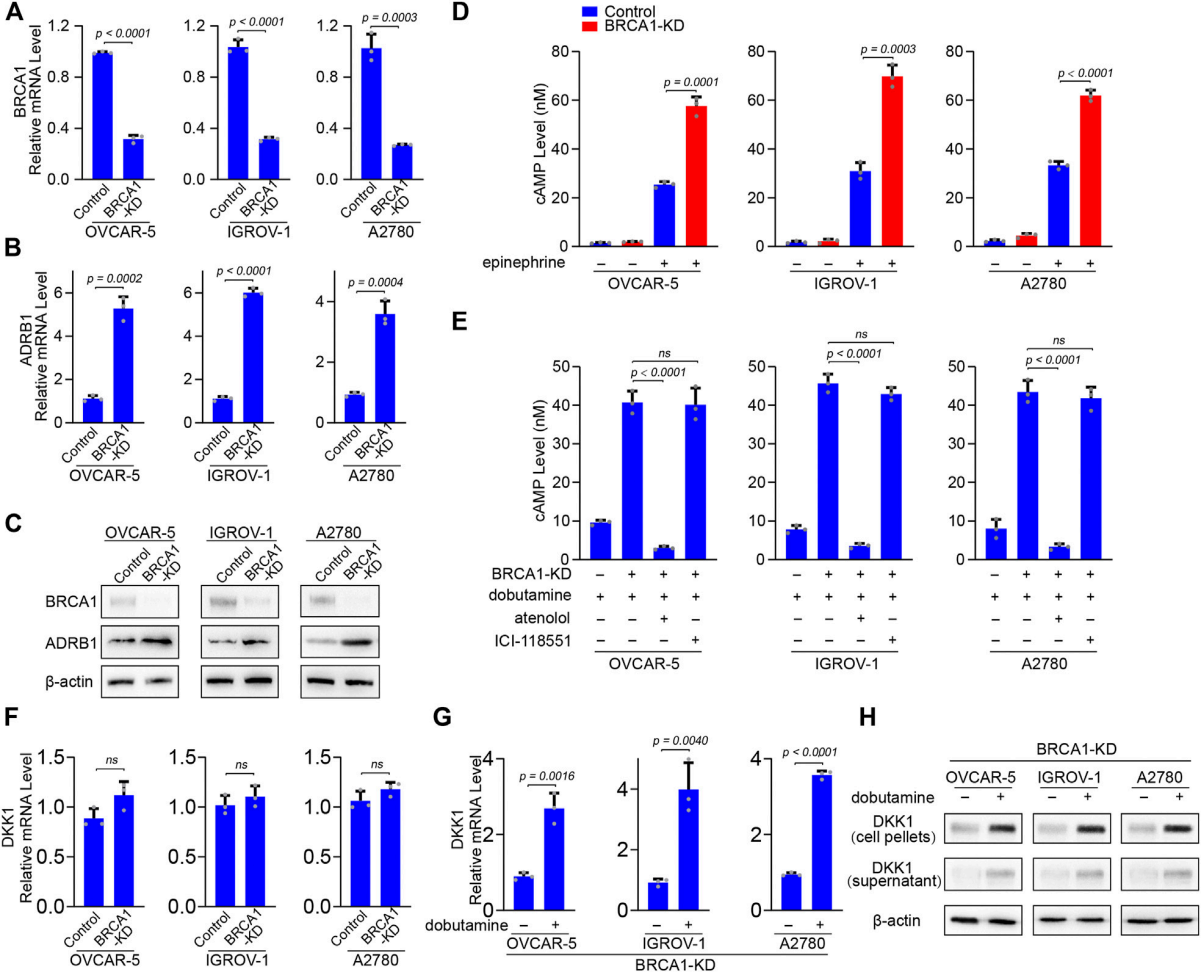
BRCA1 knock-down promotes ADRB1 expression and cAMP production in ovarian cancer cells. **(A)** qPCR analysis of BRCA1 knock-down efficacy by shRNA lentivirus in OVCAR-5, IGROV-1, and A2780 ovarian cancer cells. KD: knock down. Data are presented as mean values ±SD. *p* values were calculated by unpaired two-tailed Student’s *t*-tests. **(B)** qPCR analysis of ADRB1 mRNA expression level in control and BRCA1 knock-down OVCAR-5, IGROV-1, and A2780 ovarian cancer cells. Data are presented as mean values ±SD. *p* values were calculated by unpaired two-tailed Student’s *t*-tests. **(C)** Western blot analysis of BRCA1 and ADRB1 proteins levels in control and BRCA1 knock-down OVCAR-5, IGROV-1, and A2780 ovarian cancer cells. β-actin was used as a loading control. **(D)** cAMP levels were determined by ELISA in control and BRCA1 knock-down OVCAR-5, IGROV-1, and A2780 ovarian cancer cells treated with or without epinephrine. Data are presented as mean values ±SD. *p* values were calculated by unpaired two-tailed Student’s *t*-tests. **(E)** cAMP levels were determined by ELISA in control and BRCA1 knock-down OVCAR-5, IGROV-1, and A2780 ovarian cancer cells treated with or without dobutamine, atenolol or ICI-118551. Data are presented as mean values ±SD. *p* values were calculated by unpaired two-tailed Student’s *t*-tests. **(F)** qPCR analysis of DKK1 mRNA expression level in control and BRCA1 knock-down OVCAR-5, IGROV-1, and A2780 ovarian cancer cells. Data are presented as mean values ±SD. *p* values were calculated by unpaired two-tailed Student’s t-tests. **(G)** qPCR analysis of DKK1 mRNA expression level in BRCA1 knock-down OVCAR-5, IGROV-1, and A2780 ovarian cancer cells treated with or without dobutamine. Data are presented as mean values ±SD. *p* values were calculated by unpaired two-tailed Student’s t-tests. **(H)** Western blot analysis of DKK1 protein levels in cell pellets and supernatant from BRCA1 knock-down OVCAR-5, IGROV-1, and A2780 ovarian cancer cells treated with or without dobutamine. β-actin was used as a loading control.

We wanted to verify whether ovarian cancer cells express higher levels of ADRB1 and upon BRCA1 knock-down. We selected three ovarian cancer cell lines (OVCAR-5, IGROV-1, and A2780) bearing wild-type BRCA1 (Stordal et al., 2013) and knocked down endogenous BRCA1 using lentivirus-based shRNA. BRCA1 was successfully knocked down in the OVCAR-5, IGROV-1, and A2780 cells upon treatment with the BRCA1-targeting shRNA lentivirus, as determined by qPCR and western blot analysis (Figures 4A,C). When BRCA1 was knocked down, the mRNA and protein levels of ADRB1 in the three ovarian cancer cell lines increased compared with those in the control groups (Figures 4B,C), indicating that BRCA1 knock-down induces ADRB1 expression in ovarian cancer cells.

*In vivo*, catecholamine hormones including norepinephrine and epinephrine in the plasma can activate ADRB1, which can promote adenylyl cyclase to synthesize cAMP (Pon et al., 2016). In cultured cells, we found the level of cAMP is maintained at a relatively low concentration in the absence of stimulating factors. When stimulated ovarian cancer cells by the non-selective adrenoreceptor agonist epinephrine, the cAMP level was significantly elevated, indicating that activated adrenoreceptor can promote the production of cAMP. When BRCA1 was knocked down, cAMP generation was further increased compared with the control group after epinephrine treatment (Figure 4D). The enhanced effect on cAMP production in the BRCA1-deficient cancer cells may be due to the overexpression of ADRB1. To test this, we treated the ovarian cancer cells with the ADRB1 selective agonist dobutamine. Dobutamine treatment only induced mild cAMP production, indicating that ovarian cancer cells with normal BRCA1 maintain relatively low levels of ADRB1. However, cAMP generation dramatically increased in the ovarian cancer cells with knock-down of BRCA1 after dobutamine treatment. In addition, when we simultaneously treated the BRCA1 knock-down ovarian cancer cells with dobutamine and the ADRB1-specific antagonist atenolol, the levels of cAMP decreased to the basal level, whereas the ADRB2-specific antagonist ICI-118551 had no inhibitory effect (Figure 4E). On the other hand, we also detected the mRNA levels of DKK1 in BRCA1 knock-down ovarian cancer cell lines. No significant difference of DKK1 expression was observed between control and BRCA1 knock-down cancer cells (Figure 4F). However, when the BRCA1 knock-down cells were treated with dobutamine, the expressional level of DKK1 was dramatically elevated (Figures 4G,H). Considering DKK1 is a secreted protein, we also detected the protein level of DKK1 in the culture medium. As expected, the protein level of DKK1 in the culture medium of dobutamine-treated BRCA1 knock-down cancer cells was elevated (Figure 4H). The results demonstrate that ovarian cancer cells deficient in BRCA1 express higher levels of ADRB1, which promotes the synthesis of cAMP. The elevated cAMP further induces the expression of DKK1 in these cancer cells.

### Elevated cAMP Inhibits Apoptosis of BRCA1 Knock-Down Ovarian Cancer Cells and Proliferation of CD8^+^ T Cells

**FIGURE 5:**
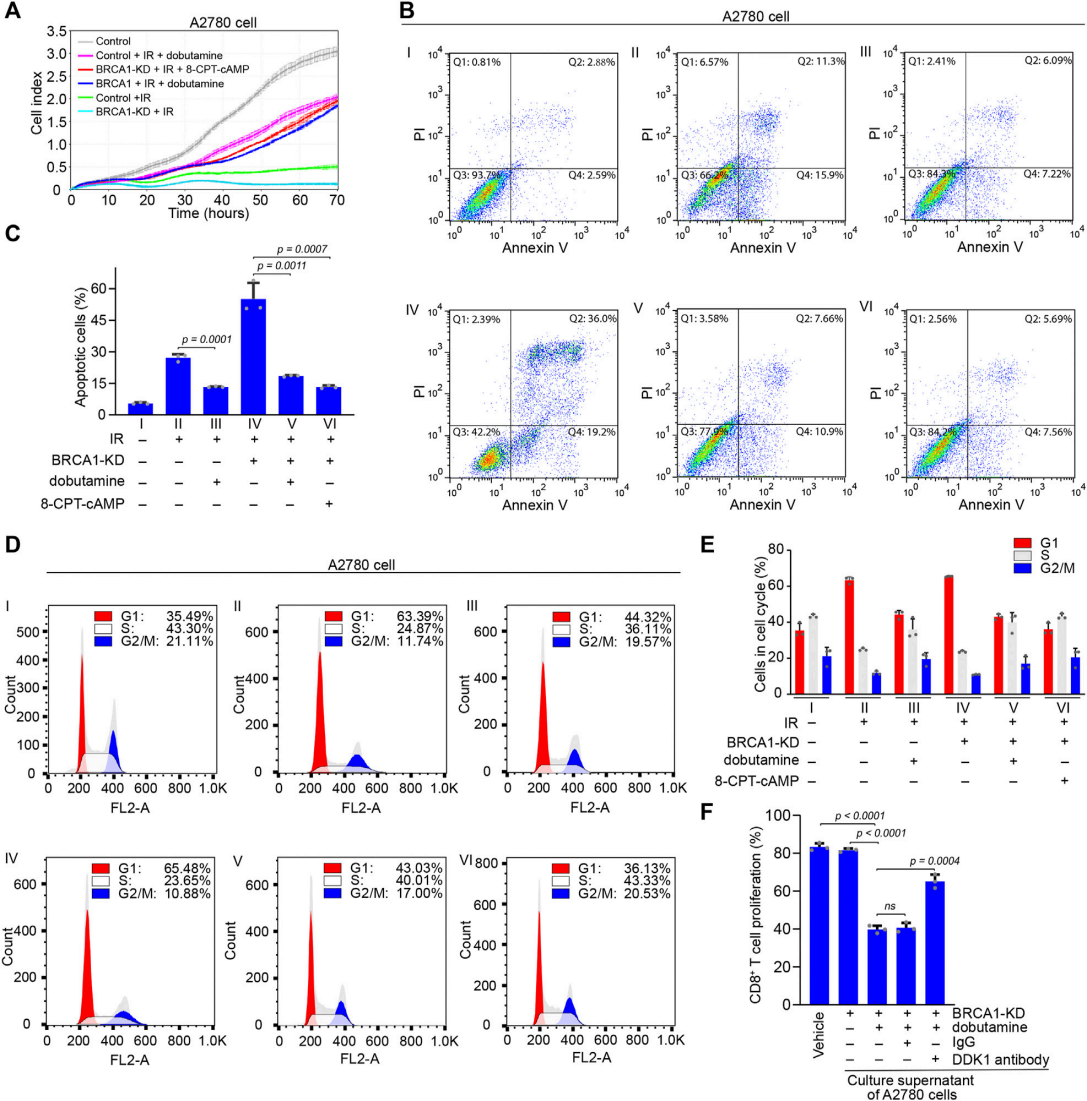
The effects of cAMP on apoptosis of BRCA1 knock-down ovarian cancer cells and proliferation of CD8^+^ T cells. **(A)** Control and BRCA1 knock-down A2780 cells were treated with or without dobutamine or 8-CPT-cAMP before IR irradiation, and the proliferation of these cells was monitored using the xCELLigence RTCA system. Cell proliferation was automatically monitored 70 h (i.e., until the control cells reached a growth plateau). Control cells are shown by grey line, IR irradiated control and BRCA1 knock-down cells are shown by green and cyan lines, dobutamine pre-treated control and BRCA1 knock-down cells irradiated by IR are shown by pink and blue lines, 8-CPT-cAMP pre-treated BRCA1 knock-down cells irradiated by IR are shown by red line. Error bars represent the standard deviation. **(B)** Control and BRCA1 knock-down A2780 cells were treated with or without dobutamine or 8-CPT-cAMP before exposed to 10 Gy IR. After 18 h, cell apoptosis analysis was performed through flow cytometry. Ⅰ: control cells without IR treatment, Ⅱ: control cells with IR treatment, Ⅲ: dobutamine pre-treated control cells with IR treatment, Ⅳ: BRCA1 knock-down cells with IR treatment, Ⅴ: dobutamine pre-treated BRCA1 knock-down cells with IR treatment, Ⅵ: 8-CPT-cAMP pre-treated BRCA1 knock-down cells with IR treatment. Q1: necrotic cells, Q2: early apoptotic cells, Q3: late apoptotic cells, Q4: viable cells. **(C)** Percentages of apoptotic A2780 cells, including early and late apoptotic cells, in each group from **(B)**. Three biologically independent replicates were performed. Data are presented as mean values ±SD. *p* values were calculated by unpaired two-tailed Student’s *t*-tests. **(D)** Cell cycle analysis was performed to detect the effects of dobutamine or 8-CPT-cAMP on cells that were treated as described in **(B)** through flow cytometry. G1 phase: red, S phase: white, G2/M phase: blue. **(E)** Percentages of G1 phase, S phase, and G2/M phase cells in each group from **(D)**. Three biologically independent replicates were performed. Data are presented as mean values ±SD. **(F)** The inhibitory effect of secretion of dobutamine pre-treated BRCA1 knock-down A2780 cells on the proliferation of CD8^+^ T cells *in vitro*. The proliferation of the cells was determined by CFSE dilution. Three biologically independent replicates were performed. Data are presented as mean values ±SD. *p* values were calculated by unpaired two-tailed Student’s *t*-tests.

To test the hypothesis that cellular cAMP suppress DNA damage-induced apoptosis in BRCA1-deficient ovarian cancer cells, we treated BRCA1 knock-down A2780 ovarian cancer cells with ionizing radiation (IR), which induces DNA double-strand breaks (Shikazono et al., 2009). After exposure to 10 Gy IR, the growth of the BRCA1 knock-down A2780 cells nearly ceased, whereas growth of IR-treated control cells was partially recovered to grow (Figure 5A). This suggests that cell cycle arrest or apoptosis induced by massive unrepaired DNA damage may lead to the arrested growth of BRCA1 knock-down A2780 cells, whereas cells with wild-type BRCA1 can recover after DNA damage repair. When A2780 cells were incubated with dobutamine or 8-CPT-cAMP (a membrane-permeable analog of cAMP) before IR treatment, the proliferation of the cells could be recovered to a large extent. As shown in Figure 5A, after incubation with dobutamine, IR-treated control and BRCA1 knock-down A2780 cells could proliferate normally. Similar to the effect of dobutamine, 8-CPT-cAMP also removed the inhibition on the BRCA1 knock-down A2780 cells proliferation. This suggests that endogenous cAMP in BRCA1 knock-down ovarian cancer cells can prevent cell death or cell cycle arrest caused by DNA damage.

Next, we investigated the apoptosis of A2780 cells after IR treatment using flow cytometry. Compared with control A2780 cells without IR treatment, which consisted of only 5.5% basal apoptotic cells, the proportions of control and BRCA1 knock-down A2780 cells that were apoptotic after IR treatment were 27.2 and 55.2%, respectively. This indicates that BRCA1 is essential for DNA damage repair, and that the lack of BRCA1 causes dramatic apoptosis in ovarian cancer cells bearing massive unrepaired DNA double-strand breaks. In contrast, in the presence of dobutamine, the percentages of control and BRCA1 knock-down A2780 cells that were apoptotic after IR treatment decreased to 13.3 and 18.5%, respectively. Similar to the effect of dobutamine, direct stimulation by 8-CPT-cAMP of IR-treated BRCA1 knock-down A2780 cells decreased the percentage of apoptotic cells to 13.25% (Figures 5B,C). At the same time, we also performed cell cycle analysis to determine the proportions of cells in each phase. The percentage of control A2780 cells in G1 phase was 35.49%. After exposure to IR, the percentages of control and BRCA1 knock-down A2780 cells in G1 phase were 63.39 and 65.48%, respectively, indicating that massive DNA damage arrested the cell cycle in G1. After dobutamine treatment, the percentages of G1 cells in IR-treated control and BRCA1 knock-down A2780 cells decreased to 44.32 and 43.03%, respectively, suggesting that cAMP terminated the cell cycle arrest caused by DNA damage. Treatment with 8-CPT-cAMP decreased the percentage of IR-treated BRCA1 knock-down A2780 cells in the G1 phase to 36.13%, a percentage similar to that of the control cells (Figures 5D,E). In addition, we tested whether DKK1 could inhibit the proliferation of CD8^+^ T cell *in vitro*. Considering that DDK1 is a secreted protein, we collected culture supernatant of BRCA1 knock-down A2780 cells that were treated with or without dobutamine. Only the secretion of dobutamine treated BRCA1 knock-down A2780 cells could inhibited the proliferation of the CD8^+^ T cells. The inhibitory effect could be compromised by adding the DKK1 antibody, but not the control IgG (Figure 5F), demonstrating that DKK1 can actually inhibit the proliferation of CD8^+^ T cells. In general, we demonstrate that cAMP promoted by ADRB1 abolishes cell cycle arrest and DNA damage induced-apoptosis in BRCA1-deficient cancer cells. The secreted DKK1 from BRCA1-deficient cancer cells on the other hand confronts immune cells, assisting the apoptotic resistance.

### cAMP Inhibits Apoptosis Through Abrogating p53 Accumulation

**FIGURE 6:**
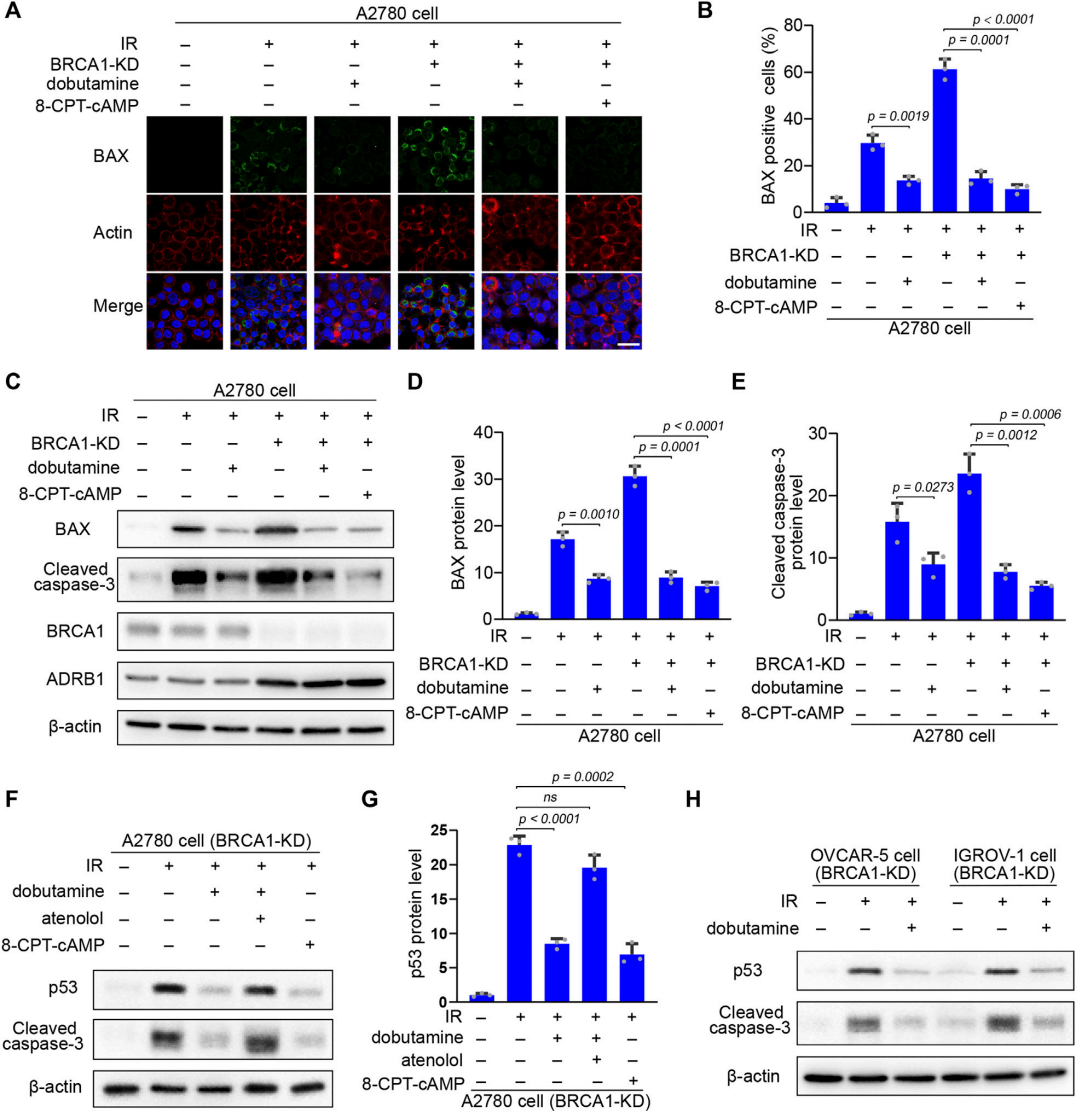
The inhibitory effect of cAMP on IR-induced apoptosis is p53 dependent. **(A)** Control and BRCA1 knock-down A2780 cells were treated as described in Panel 5C. 18 h after IR, immunofluorescence staining of pro-apoptotic pore-forming protein BAX of each cells was performed. Scale bar, 25 μm. **(B)** Percentages of BAX positive cells in each group from **(A)**. At least 100 cells were included in each group. Three biologically independent replicates were performed. Data are presented as mean values ±SD. *p* values were calculated by unpaired two-tailed Student’s *t*-tests. **(C)** Western blot analysis of BRCA1, ADRB1, BAX, and cleaved caspase-3 protein level in each group that were treated as described in **(A)**. β-actin was used as a loading control. **(D,E)** Protein levels of BAX and cleaved caspase-3 in each group from **(C)**. The relative intensities of BAX and cleaved caspase-3 bands were quantified by ImageJ. The experiments were performed three times. Data are presented as mean values ±SD. *p* values were calculated by unpaired two-tailed Student’s *t*-tests. **(F)** BRCA1 knock-down A2780 cells were treated with or without dobutamine, atenolol, or 8-CPT-cAMP before exposed to 10 Gy IR. 18 h after IR, p53 and cleaved caspase-3 protein levels in each group were determined by western blot. β-actin was used as a loading control. **(G)** Protein levels of p53 in each group from **(F)**. The relative intensities of p53 bands were quantified by ImageJ. The experiments were performed three times. Data are presented as mean values ±SD. *p* values were calculated by unpaired two-tailed Student’s *t*-tests. **(H)** BRCA1 knock-down OVCAR-5 and IGROV-1 cells were incubated with or without dobutamine before exposed to 10 Gy IR. 18 h after IR, p53 and cleaved caspase-3 protein levels in each group were determined by western blot. β-actin was used as a loading control.

Using immunofluorescence staining, we determined the level of pro-apoptotic pore-forming protein BCL-2-associated X protein (BAX) in A2780 cells. Consistent with the previous results of flow cytometry, after exposure to IR, the BAX signal in A2780 cells was significantly enhanced in comparison with that in the control cells, and the percentages of BAX-positive cells in the two groups were 29.7 and 4.1%, respectively. As expected, a stronger BAX signal was detected in the BRCA1 knock-down cancer cells upon IR irradiation, and the percentage of BAX-positive cells increased to 61.2%. If the cells were treated with dobutamine beforehand, weaker BAX signals were observed in the IR-irradiated control and BRCA1 knock-down A2780 cells, and 13.7 and 14.5% cancer cells were BAX positive, respectively. In addition, the inhibitory effect of 8-CPT-cAMP on BAX protein accumulation was similar to that of dobutamine on the IR-treated BRCA1 knock-down ovarian cancer cells (Figures 6A,B). Immunoblotting against BAX and the activated (cleaved) form of the apoptotic executioner protein caspase-3 from A2780 ovarian cancer cells further verified the above results (Figures 6C–E), confirming that the inhibitory effect of cAMP on apoptosis of ovarian cancer cells is dependent on its ability to inhibit the apoptosis pathway.

Because of the essential role of p53 in apoptosis regulation (Carneiro and El-Deiry, 2020), p53 accumulation in response to DNA damage might be inhibited by the enhanced cAMP in the BRCA1-deficient ovarian cancer cells. After IR irradiation, p53 accumulation and caspase-3 cleavage were substantially elevated in the BRCA1 knock-down ovarian cancer cells. Dobutamine or 8-CPT-cAMP treatment dramatically decreased the levels of p53 and cleaved caspase-3. However, the inhibitory effect of dobutamine on p53 accumulation and caspase-3 cleavage was abrogated by the ADRB1 selective inhibitor atenolol (Figures 6F,G). The results show that cAMP antagonism of DNA damage-induced apoptosis is dependent on the inhibition of p53 accumulation in the BRCA1-deficient ovarian cancer cells. We also examined the inhibitory effects of cAMP on apoptosis in two other ovarian cells lines (OVCAR-5 and IGROV-1) with knock-down of BRCA1. IR-induced p53 accumulation and cleavage of caspase-3 in these cancer cells could also be attenuated by dobutamine (Figure 6H). In summary, these results show the relationship between the inhibitory effect of cAMP on apoptosis and DNA damage-induced p53 accumulation, BAX induction, and cleavage of caspase-3, demonstrating that ADRB1-mediated cAMP production negatively regulates DNA damage-induced apoptosis of the BRCA1-deficient ovarian cancer cells.

## Discussion

BRCA1 is an essential homologous recombination factor that plays fundamental functions in DNA damage repair and genomic integrity maintenance (Huen et al., 2010). BRCA1 deficiency results in defective DNA damage repair and accumulation of DNA lesions that is lethal to embryos and primary mouse embryonic fibroblast cells because of the activation of p53-dependent apoptosis (Gowen et al., 1996; Hakem et al., 1996; Ludwig et al., 1997; Xu et al., 2001). However, mutations in the *BRCA1* gene dramatically increase the incidence of breast and ovarian cancers in women (Eastson et al., 1995; Rahman and Stratton, 1998; King et al., 2003). In these patients, the *BRCA1*-mutated cancer cells resist apoptosis and grow normally even their p53 is proficient. Thus, the BRCA1-deficient cancer cells may evolve some apoptotic resistance skills *in vivo*. In this study, through retrospective analysis of ovarian cancer patients’ transcriptome data, we found that ovarian cancer cells in BRCA1-deficient patients expressed higher levels of ADRB1, which can enable adenylyl cyclase to generate cAMP. Consistent with the results above, when BRCA1 was knocked down in BRCA1 wide-type ovarian cancer cell lines, the cells expressed higher levels of ADRB1, which promoted the production of cAMP. The elevated cAMP inhibited IR-induced cell apoptosis by abolishing the function of p53. On the other hand, the elevated cAMP also induced the expression of DDK1, which inhibited the proliferation of CD8^+^ T lymphocytes that can promote the immune evasion of cancer cells. When ADRB1 was inhibited, the resistance ability of BRCA1-deficient ovarian cancer cells to p53-dependent apoptosis was abrogated.

It is well known that p53 is the key factor located in the center of the complex apoptosis pathway, which can be induced by cellular stresses, such as DNA damage, cytotoxic chemicals, and oxidative stress (Aubrey et al., 2018). Under normal conditions, p53 is maintained at a low level through continuous degradation by the proteasome. Upon a cellular stress, for example, persistent or irreparable DNA damage, p53 is stabilized and aggregates in the cell nucleus, where it initiates apoptosis to clear the cells with defective genomes (Hafner et al., 2019; Carneiro and El-Deiry, 2020). Therefore, circumvention of the p53-based apoptosis response is extremely important for the tumor formation and progression, especially in BRCA1-deficient ovarian and breast cancer cells, which are prone to accumulate DNA damages. In our retrospective analysis of transcriptome data from BRCA1-deficient ovarian cancer patients, we found that the DNA damage repair-related pathways were severely attenuated, and the gene mutation frequency was much higher than that in patients with normal BRCA1, suggesting that BRCA1 deficiency caused inefficient DNA damage repair, leading to a massive number of gene mutations and extreme genome instability. However, no obvious increase in apoptotic signals was detected in the BRCA1-deficient ovarian cancer patients, indicating that apoptosis was inhibited in the BRCA1-deficient ovarian cancer cells. Of course, mutation or deletion of p53 is the most direct mechanism to resist apoptosis, but not all BRCA1-deficient cancer patients carry defective p53 (Ramus et al., 1999; Greenblatt et al., 2001; Manie et al., 2009; Jonsson et al., 2019). We found that expression of ADRB1 in BRCA1-defective ovarian cancer cells was activated by extracellular catecholamine hormones. Activated ADRB1 generates abundant cAMP, which inhibits DNA damage-induced apoptosis. Therefore, it is also possible that characteristics of the specific physiological environment in the ovarian tissue, such as the existence of extracellular survival factors (e.g., extracellular catecholamine hormones) and/or BRCA1-defective ovarian cancer cells expressing higher levels of anti-apoptosis factors (e.g., ADRB1) overwhelm the p53-activated apoptosis.

*In vivo*, adrenergic receptors on the membranes of target cells can be bound and activated by the catecholamine hormones from the plasma, and then cAMP can be utilized to activate downstream pathways that regulate the associated biological processes (Antoni et al., 2006; Paravati et al., 2018). Studies have shown that β-adrenoreceptors, especially ADRB1 and ADRB2, are highly expressed in pan cancers that significantly reduce the overall survival of tumor patients (Lehrer and Rheinstein, 2020). From this side, the poor outcomes of BRCA1-deficient ovarian cancer patients are related to anti-apoptotic ability mediated by ADRB1. In addition to ADRB1, BRCA1-deficient ovarian cancer cells also express high levels of other factors related to catecholamine-adrenoceptor-cAMP pathway regulation, such as the adenylate cyclase ADCY2 and G protein-coupled receptor ADGRB1. Considering the role of BRCA1 in regulating gene transcription (Mullan et al., 2006; Rosen et al., 2006), we speculate that BRCA1 may act as a corepressor that inhibits the transcription of these genes. Moreover, the elevated cAMP induces an increased expression of the secretory protein DKK1, which was reported to promote tumor growth and metastasis in several tumor models (Kagey and He, 2017). In our study, we found that DKK1 secreted by BRCA1-deficient cancer cells could inhibit CD8^+^ T cells proliferation, which impaired CD8^+^ T cells activation. It has been reported that enhanced serum level of DKK1 is correlated with a poor prognosis in cancer patients (Chu et al., 2021). This effect may result from the immunoregulatory role of DKK1 in generating an immunosuppressive tumor microenvironment through suppressing the proliferation of CD8^+^ T cells and other immune cells, thus facilitating the immune evasion of cancer cells. This enhances cAMP efficiency for apoptotic resistance in cancer cells. In the future, chemicals that can block the activities of these factors may be used as new therapeutic drugs against *BRCA1*-mutated tumors.

## Data Availability

Publicly available datasets were analyzed in this study. This data can be found here: https://www.cbioportal.org/study/summary?id=ov_tcga.
